# Influence of herd and environmental factors and phenotypic trends in productive and reproductive traits in dairy farms in Argentina

**DOI:** 10.1007/s11250-026-05135-1

**Published:** 2026-06-12

**Authors:** MJ Beribe, HA Carignano, M Piccardi, G Tuñon, M Bertossi, M Weber, D Pipino, C Sola, PR Marini, N López-Villalobos

**Affiliations:** 1 Instituto Nacional de Tecnología Agropecuaria (INTA), Estación Experimental Agropecuaria Pergamino (EEA Pergamino), Pergamino, Argentina; 2 Universidad Nacional de Rosario (UNR), Facultad de Ciencias Bioquímicas y Farmacéuticas (FBIOyF), Rosario, Argentina; 3 Universidad Nacional de Rosario (UNR), Programa Internacional de Investigación Lechera (PIdIL), Rosario, Argentina; 4 Instituto Nacional de Tecnología Agropecuaria (INTA), Instituto de Virología e Innovaciones Tecnológicas (IVIT), UEDD INTA – CONICET, Hurlingham, Argentina; 5 Consejo Nacional de Investigaciones Científicas y Técnicas (CONICET), Ciudad Autónoma de Buenos Aires, Buenos Aires, Argentina; 6 Instituto Nacional de Tecnología Agropecuaria (INTA), Estación Experimental Agropecuaria Oliveros (EEA Oliveros), Oliveros, Argentina; 7 Universidad Nacional de Río Cuarto (UNRC), Río Cuarto, Argentina; 8 Facultad de Ciencias Veterinarias de la Universidad Nacional del Chaco Austral (FVET - UNCAUS), Presidencia Roque Sáenz Peña, Argentina; 9 SW Agropecuaria, San Carlos, Argentina; 10 Establecimiento “El Campito”, Ucacha, Argentina; 11 Universidad Nacional de Rosario (UNR), Facultad de Ciencias Veterinarias, Casilda, Argentina; 12 School of Agriculture and Environment, Massey University, Palmerston North, New Zealand

**Keywords:** Milk yield, Fertility traits, Argentine pasture-based systems, Environmental effects

## Abstract

Argentina hosts a wide range of dairy production systems, from pasture-based to confined models. Despite this diversity, genetic improvement has relied almost exclusively on imported semen from high-merit Holstein bulls. Unregistered Holstein populations managed under pasture-based systems account for a substantial share of national milk production, yet their productive and reproductive performance remains poorly documented. To address this gap, this study evaluated the effects of herd and environmental factors on production and fertility traits and assessed recent phenotypic trends in pasture-based dairy herds. The dataset comprised 46,588 lactations from 19,936 cows across 16 commercial herds recorded between 2010 and 2023. Traits analyzed included 305-day milk, fat and protein yields, calving-to-first-service interval, days open and calving-to-calving interval. Herd, lactation number, calving month and calving year significantly affected all traits (*p* ≤ 0.0001). Peak milk yield occurred between the third and fifth lactations, while reproductive performance declined with increasing parity. Cows calving in autumn–winter (May–August) showed superior production and fertility, coinciding with months exhibiting fewer number of days with temperature–humidity index values above the heat-stress threshold. Variance component analysis indicated that cow effects explained 16–19% of the variance in production traits and 8–11% in fertility traits. Phenotypic overall trends suggested stabilization in milk yield and limited progress in fertility, although more favourable trends were observed during 2020–2023. While these recent improvements may reflect changes in herd-level conditions over time, potential genetic contributions cannot be ruled out. In pasture-based systems, the expression of imported high-merit genetics may differ across environmental conditions, underscoring the potential value of breeding programs adapted to local production conditions.

## Introduction

Holstein breed predominates in Argentine dairy systems, representing approximately 95% of the total dairy cattle population, according to a national survey (Engler et al. 2022). The total of semen doses used in those dairy farms were: Holstein 3,294,738 (93.7%), Jersey 126,569 (3.6%) and other breeds 92,515 (2.6%) (Fischman and Torres 2023). Milk production in Argentina occurs in a diverse range of systems including confined, semi-confined, and pasture-based models with varying levels of intensification. This diversity has resulted in a stratification of production models (Gimenez et al. 2022). According to the Dairy chain observatory (OCLA 2018), 50.5% of Argentine dairy farms fall into a low-production category, contributing with just 16% of the national milk output. In contrast, 3.8% of the farms produce more than 10,000 liters (L) per day and account for over 21% of total milk production. The remaining 45.8% of the herds are classified as medium-scale producers (2,000– 10,000 L/day), collectively responsible for 63% of the national volume.

Despite the diversity of production systems, the breed of cow used is essentially the same across operations. Traditional dairy breeding programs implemented over the past decades have primarily focused on improving production traits such as milk yield (Andere et al. 2017). Limited research has documented the phenotypic performance and trends of Holstein cattle managed under local dairy conditions. A similar situation exists across other Latin American countries, where the performance of specific dairy breeds under local production conditions is rarely evaluated (Rosales and Tewolde 1993; Stirling et al. 2021). In Argentina, limited genetic progress has been reported in the local officially registered Holstein population (“Holando Argentino”), milk yield increased from 5,500 to 7,900 kg/cow/year (1995–2015), with approximately 74% of this gain attributed to improvements in management and nutrition practices. Although genetic progress remained positive, its rate declined in the last decade, paralleling a plateau in phenotypic trends (Pardo et al. 2023). However, most genetic evaluations have focused solely on animals registered in breed associations, leaving the performance of unregistered cows, that contribute significantly to national milk production largely undocumented (Toledo Alvarado et al. 2014).

This study aimed to evaluate herd level and environmental factors associated with productive and reproductive performance in typical Argentine dairy herds. Based on these insights, phenotypic trends in production and reproduction traits were then analyzed for the 2010-to-2023 period. This information is intended to provide a phenotypic baseline to support future efforts to develop regionally adapted breeding programs for pasture-based dairy systems.

## Materials and methods

### Animals and dairy facilities

**Fig. 1:**
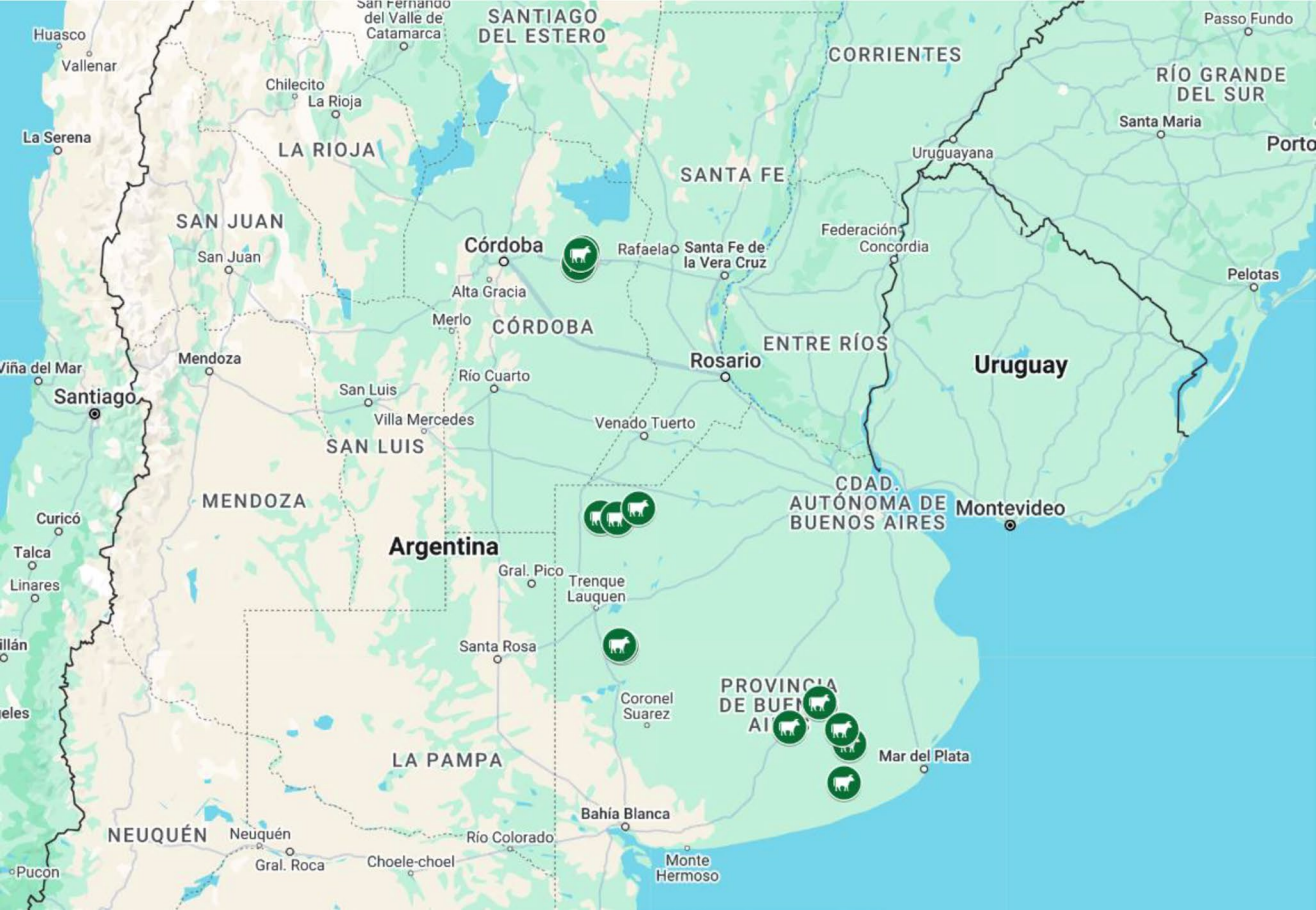
Geographic distribution of the dairy farms included in the study. Green icons denote the location of each analyzed farm

A total of 46,588 lactations from 19,936 Holstein dairy cows from 16 commercial farms located in the Argentine humid pampas region (i.e., Santa Fe, Córdoba, and Buenos Aires) (Fig. 1) were selected from the data management software SW Dr. Sola from 2010 to 2023 (Fig. 1). Herds were included based on data availability and continuity of records, without targeted selection for performance level. Therefore, the analyzed farms are considered representative of pasture-based dairy systems in the main milk-producing regions of Argentina, although they do not constitute a random or exhaustive sample. The predominant dairy production system was pasture-based with supplementation; the average diet consisted of 45% grazed pasture, 20% conserved forages (hay and silage) and 35% concentrates. Twice-daily milking was conducted on all evaluated farms.

### Dairy records description

A database containing the following production and reproduction traits was used: 305-day milk, fat, and protein yields (MY305, FY305, and PY305, respectively), adjusted using the test-interval method (Sargent et al. 1968), calving-to-first-service interval (CFSI), days open (DO), and calving-to-calving interval (CCI).

Data quality was assessed prior to the statistical analyses using descriptive statistics and visual inspection of trait distributions, including histograms and summary measures (mean, standard deviation, coefficient of variation, minimum, maximum, and proportion of missing values). Outlier detection followed biologically meaningful and distribution-based criteria. For production traits (MY305, FY305, and PY305), records outside the range mean ±3 standard deviations were excluded to remove extreme values of low frequency, representing approximately 0.26%, 0.20%, and 0.48% of the total records, respectively. For fertility traits (DO, CFSI, and CCI), extreme values above the 99th percentile were removed to limit the influence of atypical observations. In addition, based on reproductive management practices defined at the farm level, voluntary waiting periods ranging from 40 to 70 days were considered. On this basis, DO and CFSI records shorter than 35 days were considered biologically highly improbable or physiologically implausible and were excluded from the analyses. These records, together with other extreme fertility observations, likely reflect rare outliers or potential recording inaccuracies and could disproportionately influence statistical estimates if retained.

**Fig. 2:**
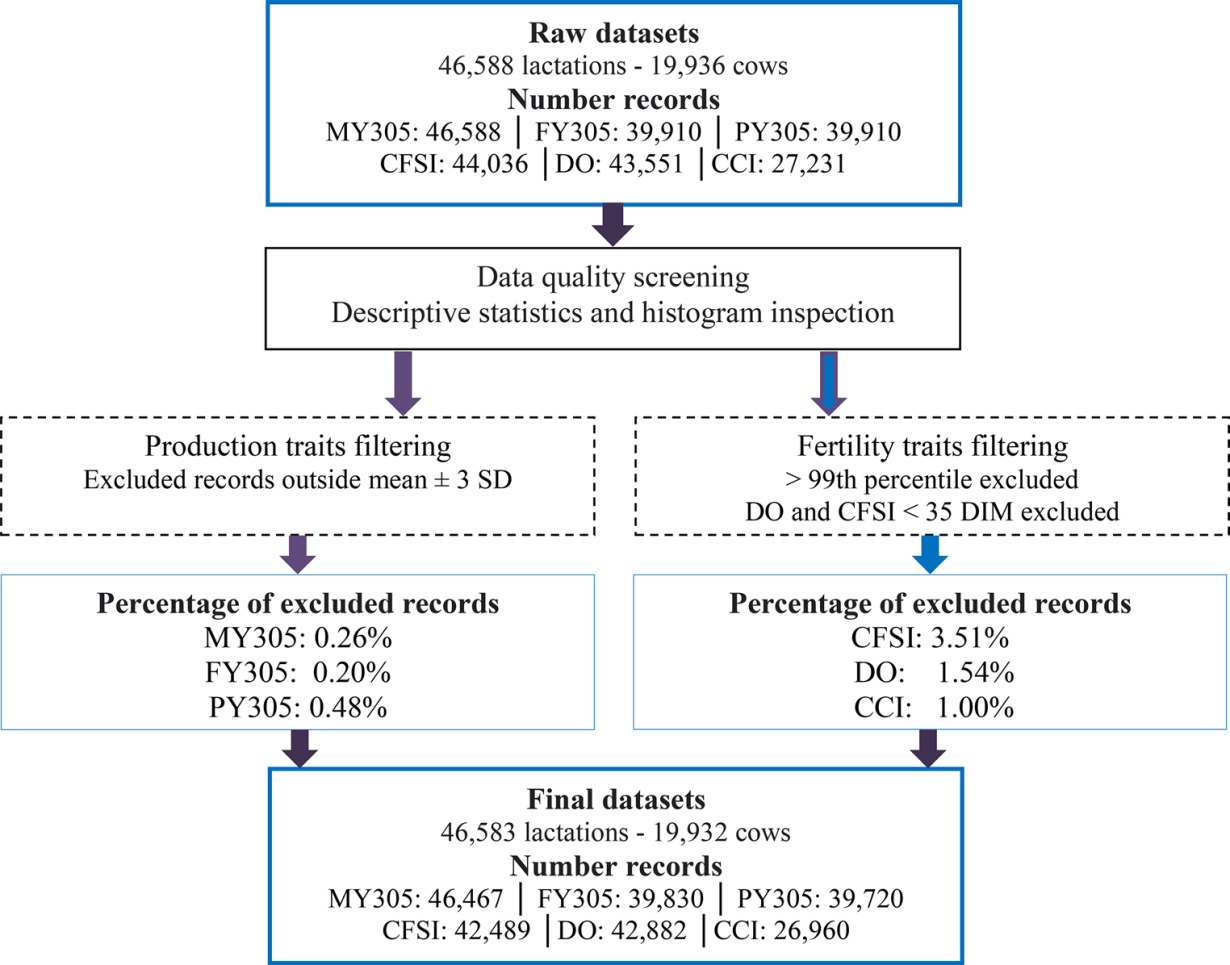
Flow diagram summarizing the data filtering procedure applied prior to statistical analyses. Filters were based on distributional criteria and biological plausibility to remove low-frequency extreme values and records considered biologically or physiologically implausible. The diagram reports the number of observations retained after each filtering step. MY305: milk yield adjusted to 305-day, FY305: fat yield adjusted to 305-day, PY305: protein yield adjusted to 305-day, CFSI: calving to first service interval, DO: Days open, CCI: calving-to-calving interval

Overall, approximately 1.54%, 3.51%, and 1.00% of records were excluded for DO, CFSI, and CCI, respectively (Fig. 2). The number of missing records and descriptive statistics for all variables after data cleaning are reported in Table 1. Because only a small proportion of records were removed and exclusions were restricted to biologically implausible observations, the overall patterns and conclusions remained robust to the applied filtering criteria.

**Table 1:** Number of records (**N**), means, standard deviations (SD), coefficient of variation (CV), minimum and maximum values of production and reproduction traits in Holstein cows from 16 commercial pasture-based herds in Argentina recorded between 2010 and 2023

**Trait**^**a**^	N	Mean	SD	CV (%)	Min	Max	Missing values (n)
MY305	46,467	8,483^*^	1,973	23	2,495	14,457	121
FY305	39,830	304^*^	78	26	70	541	6,758
PY305	39,720	282^*^	59	21	99	464	6,868
CFSI	42,489	78^+^	34	43^a^	35	275	4,099
DO	42,882	162^+^	112	69	35	600	3,706
CCI	26,960	447^+^	122	27	316	973	19,628

^a^MY305:milk yield adjusted to 305-day, FY305: fat yield adjusted to 305-day, PY305: protein yield adjusted to 305-day, CFSI: calving to first service interval, DO: days open, CCI: calving-to-calving interval

### Statistical analysis

All analyzes were performed using the statistical analysis package SAS version 9.2 (SAS Institute 2009). Descriptive statistics were obtained with the MEANS procedure. Analysis of variance for dependent variables were performed with a mixed model using the MIXED procedure (SAS Institute 2009) with the following mixed model:

$${y_{ijklm}} = \mu + {\alpha _i} + {\beta _j} + {\gamma _k} + {\delta _l} + {\tau _m} + {e_{ijklm}}$$

where:

${y_{ijklm}}$ is any of the traits evaluated: MY305, FY305, PY305, CFSI, DO and CCI

$\mu$ is the general average

${\alpha _i}$ is the fixed effect of herd i (*i* = 1, 2, … ,16),

${\beta _j}$ is the fixed effect of lactation number j (*j* = 1, 2, … ,9),

${\gamma _k}$ is the fixed effect of calving month *k* (*k* = 1, 2, … ,12),

${\delta _l}$ is the fixed effect of calving year *l* (*l* = 2010, 2011, 2012, … ,2023),

${\tau _m}$ is the random effect associated with cow m included to account for repeated measures on the same cow, and

${e_{ijklm}}$ is the random residual associated to observation ${y_{ijklm}}$.

Least squares means (LSMeans) and their standard errors were estimated for each level of the fixed effects (herd, lactation number, calving month and calving year). Pairwise comparisons among levels within each fixed effect were performed, and the resulting *p*-values were adjusted for multiple testing using the Holm procedure (Holm 1979).

Variance components were estimated for all traits using mixed models fitted with the PROC HPMIXED procedure (SAS Institute 2009). In these models, the effects of cow, herd, lactation number, calving month and calving year were included as random effects. This approach allowed partitioning of the total phenotypic variance into its corresponding variance components associated with each source of variation.

Days open (DO) were analyzed using Cox proportional hazards models with right-censored data (PROC PHREG, SAS version 9.2 (SAS Institute 2009). Cows that did not conceive were treated as right-censored at the maximum observed DO. The model included herd, lactation number, calving month, and calving year. Month-specific cumulative probabilities of conception were obtained by successive reparameterization of the model using each calving month as the reference category. Predicted probabilities of conception over time are presented as cumulative survival curves.

Daily environmental data were obtained from the National Aeronautics and Space Administration Prediction of Worldwide Energy Resources (NASA POWER) database (Stackhouse 2022), which provides gridded meteorological variables derived from satellite observations. For each herd location, daily air temperature (°C) and relative humidity (%) were extracted for the period 2010–2023. Heat stress was characterized using the temperature–humidity index (THI). For each day, maximum air temperature (T) and minimum relative humidity (RH) were derived from hourly records, and daily THI was calculated following THI = (1.8×T + 32) - (0.55–0.0055×RH) × (1.8×T − 26) (Ravagnolo et al. 2000). Days with THI > 72 were classified as heat-stress days. The number of heat-stress days was computed for each month and averaged across years.

## Results

Descriptive statistics for production and reproduction traits are shown in Table 1. MY305 had a mean of 8,483 Kg (CV = 23%), with values ranging from 2,495 to 14,457 Kg. FY305 and PY305 averaged 304 kg (CV = 26%) and 282 Kg (CV = 21%), respectively. Reproduction traits showed greater variability: CFSI averaged 78 days (CV = 43%), DO 169 days (CV = 74%), and CCI 447 days (CV = 27%). Results from the analysis of variance are presented in Table 2. All factors studied had a significant effect (*p* ≤ 0.0001) on all production and reproduction traits. The variance component analysis showed marked differences in the relative contribution of cow and herd effects between production and fertility traits (Table S1). For production traits, both cow and herd accounted for a substantial proportion of the total phenotypic variance. In MY305, FY305 and PY305, the cow effect explained approximately 16–19% of the variance, while herd effects accounted for 23–40%, reflecting considerable between-herd heterogeneity. Residual variance represented 35–45% of the total variance for production traits.

**Table 2:** F-values (first row within the factor) and *p*-values (second row within the factor) for factors affecting production and reproduction traits of pasture-based dairy cows from commercial herds of Argentina

Factor	Production trait^**a**^				Fertility trait^**a**^		
	MY305	FY305	PY305	CFSI		DO	CCI
Herd	932.72 (<.0001)	1,775.77 (<.0001)	692.93 (<.0001)	306.93 (<.0001)		100.68 (<.0001)	77.22
							(<.0001)
Lactation number	444.76 (<.0001)	300.61 (<.0001)	312.26 (<.0001)	6.39 (<.0001)		93.00 (<.0001)	16.51
							(<.0001)
Calving-month	72.79 (<.0001)	38.91 (<.0001)	34.50^a^(<.0001)	130.43 (<.0001)		36.57 (<.0001)	27.70
							(<.0001)
Calving-year	145.79 (<.0001)	246.73 (<.0001)	236.69 (<.0001)	25.60 (<.0001)		56.12 (<.0001)	23.82
							(<.0001)

^a^MY305:milk yield adjusted to 305-day, FY305: fat yield adjusted to 305-day, PY305: protein yield adjusted to 305-day, CFSI: calving to first service interval, DO: days open, CCI: calving-to-calving interval

In contrast, fertility traits (CFSI, DO and CCI) were dominated by residual variance, which accounted for 70–80% of the total phenotypic variance. The contribution of cow and herd effects to fertility traits was comparatively modest, with cow effect explaining 8–11% and herd effect 5–16% of the variance, indicating a greater influence of unmeasured factors on reproductive performance.

As shown in Table 3, average production traits varied significantly among the studied herds (T). Pairwise comparisons adjusted using the Holm procedure revealed that herds T1, T2 and T4 exhibited the highest mean MY305, whereas herd T16 had the lowest, with a difference of 3,814 Kg between these extreme herds. For FY305, T1 and T2 also recorded the highest mean fat yield, while T16 had the lowest. In contrast, PY305 was highest in T3 and lowest again in T16. The patterns observed for FY305 and PY305 closely mirrored MY305 yield. However, when expressed as percentages, fat content was identical (3.7%) in both the highest- and lowest-yielding herds, while protein content was slightly higher in the lowest-yielding herd (3.4%) compared with the highest (3.1%).

**Table 3:** Least squares means (± standard errors) for production and fertility traits, of purebred Holstein cows from pasture-based commercial herds of Argentina, by herds

Herd	N	Production trait^a^					Fertility trait^a^		
		MY305	FY305	PY305	CFSI			DO	CCI
		(kg)	(kg)	(kg)	(days)			(days)	(days)
T1	3,619	9,486^A^±43.9	352^A^±1.6	296^AB^±1.4	80^CDE^±0.8			203^BCD^±2.8	476^CD^ ±3.6
T2	3,636	9,350^A^±54.9	338^BCD^±2.0	291^BCD^±1.7	798^DEGF^±1.0			209^B^±3.5	484^C^±4.9
T4	3,754	9,303^AB^±61.5	338^BCD^ ±2.2	288^CDE^±1.9	75^GH^±1.1			207^BC^±3.9	484^C^±5.6
T3	1,788	9,269^B^±43.8	342^BC^±1.6	399^A^±1.4	83^BC^±0.8			200^BCDE^±2.8	464^DEF^ ±3.8
T5	1,338	9,173^BC^±43.3	341^BC^±1.6	288^DEF^±1.4	76^GH^±0.8			195^EFG^±2.8	469^CDE^±3.7
T6	5,870	9,114^BC^±46.6	333^CD^±1.7	284^EF^±1.5	76^H^±0.9			205^BCD^±3.0	477^CD^ ±4.0
T7	2,975	9,050^C^±38.9	341^BC^±1.5	292^C^±1.3	80^DEF^±0.8			198^CDEF^±2.7	460^EFG^±3.4
T8	2,730	8,258^D^±48.2	267^EF^±2.0	277^GH^±1.8	76^GH^±0.9			178^H^±3.0	440^H^^I^±3.7
T10	4,440	8,123^D^±56.6	257^G^±2.1	279^H^±1.6	77^EFGH^±1.0			184^GH^±3.6	437^FG^ ±4.6
T9	1,587	8,106^D^±41.6	273^EF^±1.8	272^FG^±1.8	78^FGH^±0.8			177^H^±2.7	437^I^±3.2
T11	1,525	7,871^E^±48.3	244^H^±1.5	265^I^±1.5	84^BC^±0.9			193^EFG^±3.1	453^FGH^ ±3.8
T12	2,470	7,805^EG^±40.9	260^FG^±1.8	263^I^±1.3	84^B^±0.8			190^FG^±2.6	451^GH^ ±3.2
T13	2,770	7,686^G^±45.9	232^I^±1.9	257^J^±1.7	82^BCD^±0.9			191^EFG^±3.0	441^HI^±3.9
T14	4,379	7,660^G^±56.9	NR	NR	113^A^±1.1			257^A^±3.8	535^A^±4.9
T15	2,175	6,250^H^±50.6	221^J^±1.9	213^K^±1.6	113^A^±0.9			254^A^±3.3	415^B^±4.0
T16	1,411	5,672^I^±63.3	209^K^±2.3	199^L^±1.9	116^A^±1.1			259^A^±4.0	512^B^±4.9

^a^MY305:milk yield adjusted to 305-day, FY305: fat yield adjusted to 305 day, PY305: protein yield adjusted to 305-day, CFSI: calving to first service interval, DO: days open, CCI: calving-to-calving interval. NR: Not recorded.

^A, B,...K^Means within a column with different superscript letters indicate statistically significant differences (P < 0.05) based on pairwise comparisons with Holm adjustment. Red letters denote the highest values, while green letters indicate the lowest values within each comparison

Fertility also varied among herds; herds T16, T15 and T14 exhibited the longest average CFSI, while T4 and T6 showed the shortest intervals. Similarly, T16, T15 and T14 had the highest average days open (DO), whereas the shortest DO values were observed in herds T8, T9 and T10. Regarding CCI, herds T16, T15 and T14 again showed the longest intervals, whereas the shortest CCI values were observed in herds T8 and T9.

Production performance differed significantly across lactation numbers (Table 4). Highest MY305 yields were observed during the 3rd and 4th lactation, which were also associated with higher FY305 and PY305 yields. Notably, the 9th lactation had similar MY305, FY305, and PY305 to those of the 1st lactation.

**Table 4:** Least squares means (± standard errors) for production and fertility traits, of purebred Holstein cows from pasture-based commercial herds of Argentina, by lactation number

Lactation number	N	Production trait^a^				Fertility trait^a^		
		MY305	FY305	PY305	CFSI		DO	CCI
		(kg)	(kg)	(kg)	(days)		(days)	(days)
1	17,521	7,741^**D**^ ± 13.8	276^**F**^ ±0.5	262^E^ ± 0.5	83^B^ ± 0.3		153^**F**^ ± 0.9	N/A
2	12,839	8,545^B^ ± 15.3	302^B^ ± 0.6	286^B^ ± 0.5	82^**C**^ ± 0.3		166^**E**^ ± 1.1	447^**D**^ ± 1.2
3	8,086	8,777^A^± 18.5	309^A^ ± 0.7	289^A^ ± 0.6	82^B**C**^ ± 0.4		181^D^ ± 1.3	460^C^ ± 1.4
4	4,354	8,762^A^ ± 24.4	307^A^ ± 0.9	287^B^ ± 0.8	84^B^ ± 0.5		195^C^ ± 1.8	463^BC^ ± 1.9
5	2,103	8,542^B^ ± 34.2	297^C^ ± 1.4	279^C^ ± 1.2	82^B^ ± 1.1		209^B^ ± 2.5	468^ABC^ ± 2.7
6	975	8,363^C^ ± 49.4	291^CDE^ ± 1.9	269^D^ ± 1.7	81^B**C**^ ± 1.1		217^B^ ± 3.7	478^A^ ± 3.9
7	429	8,226^C^ ± 74.2	285^DE^ ± 2.9	267^DE^ ± 2.6	91^A^ ± 1.6		237^A^ ± 5.6	484^A^ ± 5.8
8	184	7,879^**D**^ ±112.7	272^**EF**^ ± 4.5	250^**FG**^ ± 3.9	90^AB^ ± 2.5		251^A^ ± 8.4	488^AB^ ± 8.8
9	97	7,51^**D**^ ± 166.1	269^**EF**^ ± 6.5	243^**G**^ ± 5.6	98^A^ ± 3.5		247^A^ ± 11.8	478^AB^ ± 12.4

^a^MY305:milk yield adjusted to 305-day, FY305: fat yield adjusted to 305-day, PY305: protein yield adjusted to 305-day, CFSI: calving to first service interval, DO: days open, CCI: calving-to-calving interval. N/A: Not applicable.

^A, B,... F^ Means within a column with different superscript letters indicate statistically significant differences (P < 0.05) based on pairwise comparisons with Holm adjustment. Red letters denote the highest values, while green letters indicate the lowest values within each comparison

Reproductive performance declined progressively with increasing lactation number. The CFSI remained relatively stable (~81–84 days) through the first 6th lactations but increased significantly from the 7th lactation onwards, reaching approximately 98 days in the 9th lactation (*p* < 0.05). A similar trend was observed for DO, which rose steadily from around 153 days in first-lactation cows to over 247 days by the 9th lactation. CCI increased gradually from 447 days in the 2nd lactation to a peak of 488 days in the 8th lactations.

MY305 yield differed largely according to calving month, with the highest mean MY305 observed in cows calving between May and August (8,482–8,540 kg), which were significantly greater than those recorded for cows calving in December, January and February (7,815–8,019 kg). A similar pattern was observed for fat and protein yields, with peak FY305 (300 ± 1.3 kg) and PY305 (280 ± 1.1 kg) occurring in May, significantly surpassing the lowest values observed in January and December for both traits (Table 5).

**Table 5:** Least squares means (± standard errors) for production and fertility traits, of purebred Holstein cows from pasture-based commercial herds of Argentina, by calving month

Calving month	N	Production trait^a^				Fertility trait^a^		
		MY305	FY305	PY305	CFSI		DO	CCI
		(kg)	(kg)	(kg)	(days)		(days)	(days)
Jan	2,160	7,815^**G**^ ± 43.6	277^**G**^ ± 1.7	259^**G**^ ± 1.5	93 ^AB^±0.9		214^AB^ ± 3.1	495^A^ ± 3.9
Feb	4,193	7,964^**F**^ ± 36.7	283^**FG**^ ± 1.4	266^**F**^ ± 1.2	96^A^ ± 0.8		201^**D**^ ± 2.6	477^BC^ ± 3.2
Mar	5,569	8,113^E**F**^ ± 34.6	285^EF**G**^ ± 1.3	269^E^ ± 1.2	84^D^ ± 0.7		190^**E**^ ± 2.4	469^CD^ ± 3.0
Apr	5,367	8,323^C^ ± 34.9	294^CDE^ ± 1.4	275^BC^ ± 1.2	80^**E**^ ± 0.7		188^**E**^ ± 2.4	464^D**E**^ ± 3.0
May	5,328	8,540^A^ ± 35.2	299^ABC^ ± 1.4	279^A^ ± 1.2	81^**E**^ ± 0.7		191^**E**^ ± 2.5	456^**EF**^ ± 3.1
Jun	4,978	8,518^AB^ ± 35.6	298^ABCD^ ± 1.4	276^AB^ ± 1.2	80^**E**^ ± 0.7		200^**D**^ ± 2.5	453^**F**^ ± 3.1
Jul	4,670	8,523^A^ ± 36.1	296^ABCD^ ± 1.4	275^BC^ ± 1.2	80^**E**^ ± 0.7		205^C**D**^ ± 2.5	450^**F**^ ± 3.2
Aug	4,047	8,482^AB^ ± 37.3	295^BCD^ ± 1.5	275^BC^ ± 1.3	79^**E**^ ± 0.8		213^B^ ± 2.6	463^DE^ ± 3.4
Sep	3,135	8,414^BC^ ± 39.6	292^CDE^ ± 1.5	273^CD^ ± 1.3	83^D^ ± 0.8		222^A^ ± 2.8	474^BCD^ ± 3.7
Oct	2,467	8,293^C^ ± 42.2	290^DE**F**^ ± 1.7	270^DE^ ± 1.4	90^C^ ± 0.9		218^AB^ ± 3.0	474^BCD^ ±3.9
Nov	2,281	8,126^DE^ ± 43.2	286^a^^E**FG**^±1.7	266^E**F**^ ± 1.5	92^BC^ ± 0.9		220^AB^ ± 3.0	486^AB^ ± 4.0
Dec	2,272	8,019^E**F**^ ± 43.0	282^**FG**^ ± 1.7	263^**FG**^ ± 1.5	95^A^ ± 0.9		212^BC^ ± 3.1	491^A^ ± 3.8

^a^MY305:milk yield adjusted to 305-day, FY305: fat yield adjusted to 305-day, PY305: protein yield adjusted to 305-day, CFSI: calving to first service interval, DO: days open, CCI: calving-to-calving interval.

^A, B,... H^Means within a column with different superscript letters indicate statistically significant differences (P < 0.05) based on pairwise comparisons with Holm adjustment. Red letters denote the highest values, while green letters indicate the lowest values within each comparison

Reproductive performance also showed statistically significant differences depending on calving month; CFSI was longer for cows calving in February and December (96 and 93 days, respectively), and significantly shorter in May through August (79 to 81 days). The DO trait was shortest from March to July (188–200 days), and longest from September to January (214–222 days), whereas CCI had the longest intervals recorded in January, November, and December (486–495 days), and the shortest in April and August (453–463 days) (Table 5).

**Fig. 3:**
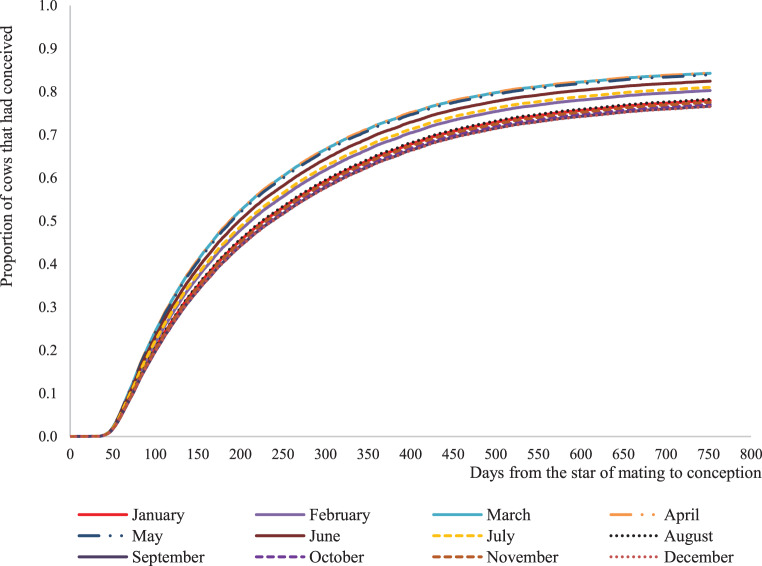
Predicted cumulative probability of conception as a function of DO, according to calving month. Predictions were adjusted for herd, lactation number, and calving year

**Fig. 4:**
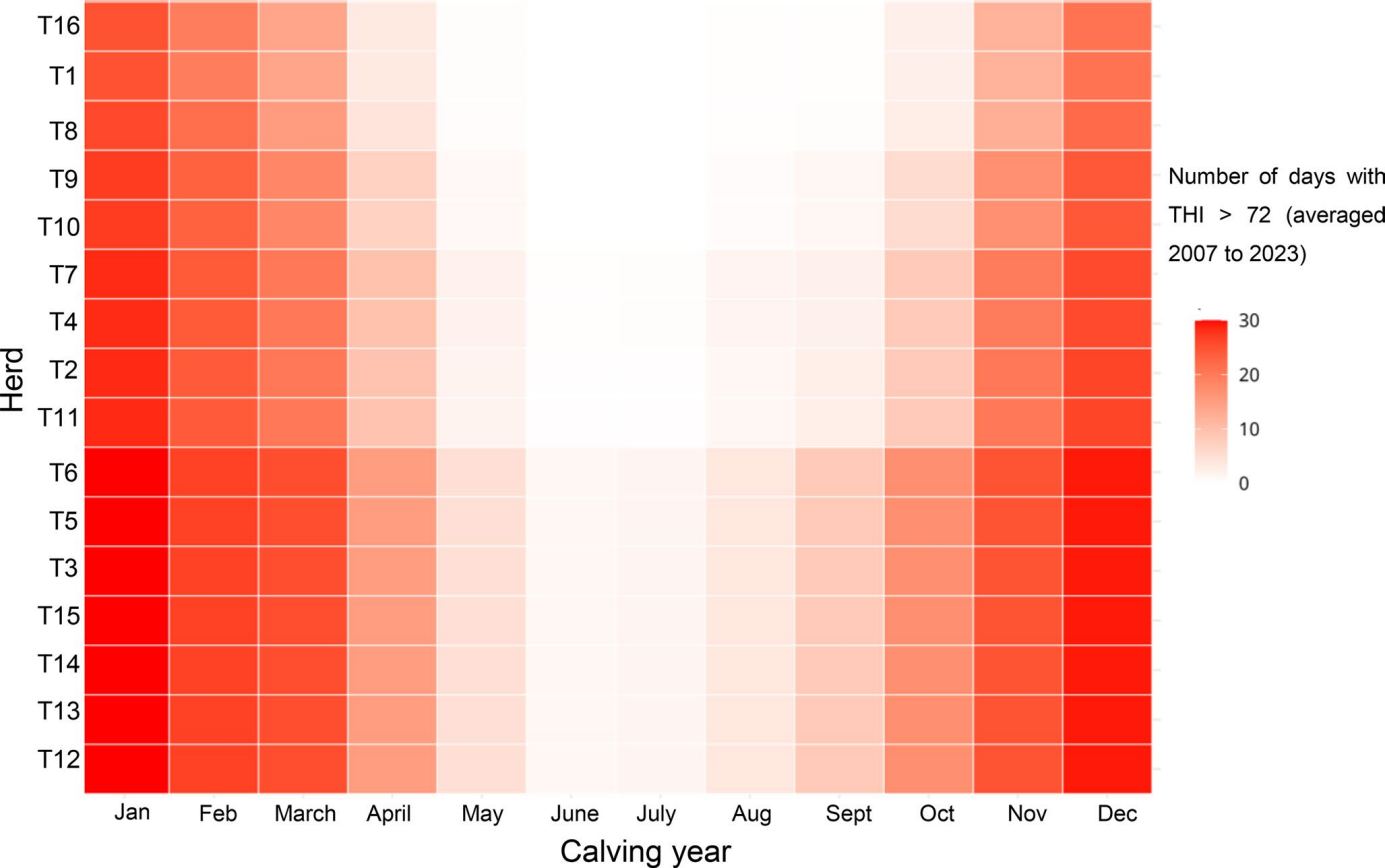
Number of days per month with temperature–humidity index (THI) values exceeding 72, averaged across years (2010–2023)

To complement these analyses, we performed a survival analysis of DO, all covariates included in the previous model were also statistically significant (*p* < 0.0001). Interestingly, cows calving in April, March and May showed the fastest median time to conception (50% cumulative probability of conception) at 166, 167 and 168 days, respectively, indicating shorter DO (Fig. 3). Whereas cows calving in January, November, October, September and December exhibited the slowest increase in the cumulative probability of conception, 198, 200, 204, 205 and 207, respectively, reflecting longer intervals to pregnancy (Fig. 3). The monthly differences in productive and reproductive performance seems consistent with seasonal patterns of heat stress exposure. As shown in Fig. 4, the months corresponding to autumn-winter calvings (April - August), coincide with the lowest number of days (averaged across 2010–2023) with THI exceeding the heat stress threshold (THI > 72). In contrast, spring/summer months (October–February), showed poorer reproductive indicators, were characterized by the highest frequency of days above this threshold.

Table 6 presented production performance according to calving year, although some improvements were observed in recent years, particularly between 2020 and 2023. Mean MY305 yield fluctuated over years, with the lowest values recorded in 2012 (7,890 ± 38.9 kg) and 2016 (7,823 ± 36.9 kg), and the highest in 2023 (9,029 ± 54.5 kg). Similar high values were already reached in 2021 (8,854 ± 38.7 kg) and 2022 (8,869 ± 39.1 kg), suggesting stabilization of yields rather than a continued upward growth. Means for FY305 and PY305 reached their peak in 2023 (322 ± 2.1 kg and 304 ± 1.8 kg, respectively), but levels close to these were already recorded from 2020 thereafter.

**Table 6:** Least squares means (± standard errors) for production and fertility traits, of purebred Holstein cows from pasture-based commercial herds of Argentina, by calving year

Calving year	N	Production trait^a^				Fertility trait^a^		
		MY305	FY305	PY305	CFSI		DO	CCI
		(kg)	(kg)	(kg)	(days)		(days	(days)
2010	2,193	8,016^FG**H**^ ±45.2	285^DE^ ± 1.7	263^F^ ± 1.5	89^AB^ ± 0.9		217^BCDE^ ± 3.1	474^BCDE^ ± 4.0
2011	2,671	8,241^D^ ± 41.8	292^C^ ± 1.6	274^D^ ± 1.4	87^BCD^ ± 0.8		209^E^ ± 2.8	476^BCD^ ± 3.6
2012	3,125	7,890^**HI**^ ± 39.8	285^DE^ ± 1.6	257^G^ ± 1.4	92^A^ ± 0.8		226^AB^ ± 2.7	471^DE^ ± 3.5
2013	3,913	8,090^EF^ ± 37.3	286^DE^ ± 1.5	266^EF^ ± 1.3	87^BC^ ± 0.8		226^A^ ± 2.6	485^AB^ ± 3.3
2014	4,573	8,038^FG^ ± 35.8	271^**F**^ ± 1.4	251^**I**^ ± 1.2	86^BCD^ ± 0.7		219^ABCD^ ± 2.5	481^ABCD^ ± 3.2
2015	4,715	8,150^DE^ ± 35.4	280^E^ ± 1.4	253^G**H**^ ± 1.2	88^B^ ± 0.7		221^ABC^ ± 2.5	489^A^ ± 3.1
2016	3,917	7,823^**I**^ ± 36.9	259^**G**^ ± 1.5	253^**HI**^ ± 1.3	88^B^ ± 0.8		213^DE^ ± 2.6	484^AB^ ± 3.3
2017	3,378	8,070^EFG^ ±37.4	274^**F**^ ± 1.5	268^E^ ± 1.3	86^BCD^ ± 0.8		221^ABCD^ ± 2.7	481^ABC^ ± 3.4
2018	3,326	7,972^G**H**^ ± 38.3	284^DE^ ± 1.5	264^F^ ± 1.3	87^BCD^ ± 0.8		213^CDE^ ± 2.8	476^BCDE^ ± 3.5
2019	3,144	8,001^FG**H**^ ± 39.2	282^DE^ ± 1.5	264^F^ ± 1.3	81^**E**^ ± 0.8		194^**F**^ ± 2.8	471^CDE^ ± 3.6
2020	3,596	8,610^C^ ± 37.9	310^B^ ± 1.5	288^C^ ± 1.3	79^**E**^ ± 0.8		186^**F**^ ± 2.7	461^E**F**^ ± 3.5
2021	3,321	8,854^B^ ± 38.7	312^B^ ± 1.5	287^C^ ± 1.3	84^**D**^ ± 0.8		191^**F**^ ± 2.7	448^**G**^ ± 3.4
2022	3,228	8,869^AB^ ± 39.1	312^A^ ± 1.5	292^Ba^ ± 1.3	85^C**D**^ ±0.8		186^**F**^ ± 2.8	449^**FG**^ ± 3.5
2023	1,170	9,029^A^ ± 54.5	322^A^ ± 2.1	304^A^ ±1.8	86^BC**D**^ ± 1.1		165^**G**^ ± 4.2	444^**FG**^ ± 5.2

^a^MY305:milk yield adjusted to 305-day, FY305: fat yield adjusted to 305-day, PY305: protein yield adjusted to 305-day, CFSI: calving to first service interval, DO: days open, CCI: calving-to-calving interval.

^A, B, ...J^Means within a column with different superscript letters indicate statistically significant differences (P < 0.05) based on pairwise comparisons with Holm adjustment. Red letters denote the highest values, while green letters indicate the lowest values within each comparison

Fertility traits showed minor fluctuations, CFSI ranged ranged between 86 and 92 days during 2010–2018. Similarly, DO decreased in some years, reaching a minimum in 2023 (165 ± 4.2 days). The CCI also decreased after 2015 but appeared to stabilize around 444–461 days between 2020 and 2023.

**Fig. 5:**
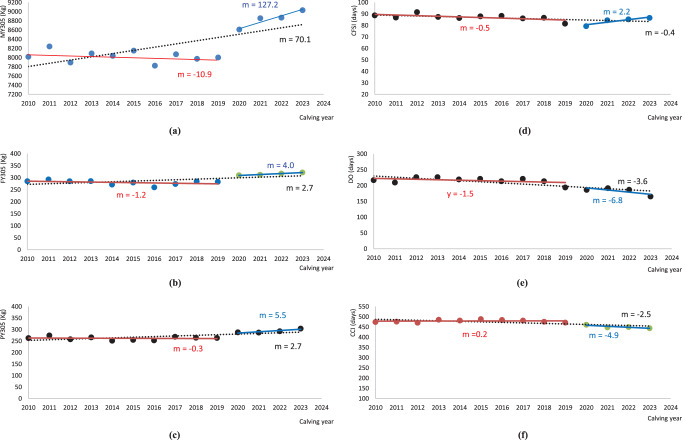
Temporal trends in least squares means of production and fertility traits by calving year. Panels (**a**–**c**) show production traits (MY305, FY305, PY305), and panels (**d**–**f**) fertility traits (CFSI, DO, CCI). Dashed lines indicate overall simple linear regressions fitted for 2010–2023, whereas solid lines indicate period-specific regressions for 2010–2019 (red) and 2020–2023 (blue). The slopes (**m**) indicate the average annual change (kg/year or days/year)

Overall trends suggest a plateau rather than sustained progress in phenotypic performance, however to further characterize temporal patterns, linear regressions of least squares means for production and fertility traits on calving year were estimated. When evaluated over the entire period, slopes (m) for production (kg/calving year) (m_MY305_ = 70.1, m_FY305_ = 2.7, m_PY305_ = 2.7) and fertility (days/calving year) (m_CFSI_ = −0.4, m_DO_ = −6.8, m_CCI_ = −4.9) were small, supporting the interpretation of overall stabilization (Fig. 5). However, when separate slopes were estimated for earlier (2010–2019) and more recent years (2020–2023), a clearer pattern emerged. Specifically, MY305 showed a weak negative slope (m_MY305_ = −10.9) from 2010 to 2019, followed by a marked positive slope (m_MY305_ = 127.2) from approximately 2019 onwards (Fig. 5), indicating a renewed increase in production in the most recent years. A similar pattern to that observed for MY305 was detected for FY305 and PY305 (Fig. 5), with shallow slopes during 2010–2019 followed by more pronounced positive slopes between 2020 and 2023. Fertility traits exhibited distinct but complementary temporal patterns. DO showed a clear declining trend over time, with a negative slope (m_DO_ = −1.5) during 2010–2019 followed by a steeper decrease from 2020 (m_DO_ = −6.8) (Fig. 5). The CCI declines also became more pronounced in the later years (m_CCI_ = −4.9). In contrast, CFSI remained relatively stable, showing only a slight increase in the most recent years (m_CFSI_ = 2.2) (Fig. 5). Recent improvements in conception efficiency translated into shorter CCI despite the relative stability of CFSI.

## Discussion

The increase in US dairy productivity has been driven by a long-term, structured genetic improvement program that began over a century ago with the implementation of milk production testing. This foundation was strengthened by subsequent advancements such as pedigree recording, progeny testing, the development of frozen semen technology, and widespread adoption of artificial insemination. However, traditional genetic improvement strategies were constrained by the high cost and lengthy duration of progeny testing (Weigel et al. 2017). In 2008, the advent of genomic selection based on molecular markers revolutionized this process by significantly reducing the generation interval and enhancing the reliability of predicted breeding values, thereby accelerating genetic gains in dairy populations (Hayes et al. 2009; Wiggans et al. 2011). Nowadays, milk production per cow in the United States averaged 11,000 kg for 2024 (/http://www.nass.usda.gov/). The global replacement of native dairy breeds with US Holsteins has been largely driven by their superior potential for milk yield (Hansen 1999). Nevertheless, typical management on a US dairy farm use confinement system, especially freestall barns which provide individual stalls and shelter, where the feeding strategy is based on a total mixed ration, a fully blended diet composed of forages, grains, by-products, and supplements, delivered once or twice a day (Endres and Espejo 2010). The dairy producers around the world have had access to high-ranking US genomic sires. However, the introduction of such germplasm into local production systems often overlooks potential genotype × environment interactions, raising concerns about long-term adaptation and whether imported sires are truly being realized under the specific conditions of pasture-based systems. This study provides a long-term phenotypic characterization of production and reproductive performance in pasture-based dairy herds in Argentina with Holstein cows. Variance component analysis showed that cow effects explained only a limited proportion of the total variance, particularly for fertility traits, indicating that a large proportion of phenotypic variability remains attributable to herd-level and residual effects. However, the present study relied on phenotypic data, the relative contribution of genetic and environmental factors could not be disentangled, thereby limiting the ability to establish causal relationships among the variables analyzed. Marini et al. (2015) reported that the use of Holstein bull semen selected in the United States has disrupted locally adapted populations commonly used in pasture-based dairy systems. Their findings emphasize the significance of genotype × environment interactions and support the need to limit the use of imported semen from bulls selected under intensive confinement systems with high levels of supplementation. Such genetics may not perform optimally under local conditions, which rely predominantly on year-round grazing (Gastaldi et al. 2020), albeit with higher supplementation levels than those typically found in countries like Ireland or New Zealand (Lazzarini et al. 2019). Conversely, Fischman and Torres (2023) noted that the belief still persists among importers, sellers, and advisors that the genetic gains reported in the countries of origin of these bulls are readily transferable to Holstein herds in Argentina. The 305-day milk yields (Table 1) were comparable to those reported by Schultz et al. (2023) for cows in Argentine dry-lot systems, despite the fact that herds in this study were pasture-based systems (with at least 50% of dry matter intake derived from pastures or forage crops). These results indicate that comparable phenotypic levels of milk production can be achieved under pasture-based systems despite differences in feeding strategies and management conditions and may question the rationale for shifting toward intensive systems when milk yield differences may not be substantial.

The average CFSI values remain far from the optimal 60 days, and DO values similarly exceed the recommended 82 days to achieve one calving per year (Holmes and MacMillan 1982). The lowest-yielding farm also exhibited the highest CFSI and DO values. These results are consistent with those of Marini et al. (2011), who reported Holstein cows producing either 9,500 or 6,500 L of milk with lower reproductive efficiency in the high-producing groups. However, the CFSI (63–93 days) and DO (81–221 days) ranges reported in that study were narrower than those found in the present work. The significant herd effect observed for both productive and reproductive traits likely reflects a composite efffect encompassing multiple herd-level factors, including management practices, nutritional strategies, environmental conditions and herd structure. The telative contribution of its individual components cannot be separately quantified in the presen study.

The production values for MY305, FY305 and PY305 according to lactation number, follow the expected pattern, with peak of production occurring between the third and fifth lactations, followed by a decline (Jingar et al. 2014; Salfer et al. 2019). For the reproductive traits, the steady increase in DO and CFSI beyond the 6th lactation emphasizes the difficulty in maintaining optimal fertility as cows advance in parity, evidencing an age-related physiological decline and/or accumulated management challenges affecting reproductive performance in cows.

The month of calving is a key factor in seasonal pasture-based systems, as it determines the alignment between nutritional demands and forage quality and availability (O‘callaghan and Boland 1999). In the present study, cows calving during the autumn-winter months (May to August) demonstrated significantly superior performance in both production and fertility traits. These months also coincided with the most favourable reproductive indicators, with CFSI values around 79–81 days, DO near 200 days, and shorter CCI of 456–463 days. Survival analysis of DO further supported the seasonal pattern observed, as cows calving in autumn months reached 50% probability of conception earlier than those calving in spring–summer, a difference that is consistent with long-term patterns of heat stress exposure, given that months with shorter DO coincided with those with fewer number of days (averaged 2010–2023) exceeding the THI threshold associated with heat stress. In contrast, cows calving during the summer months (December to February) showed significantly lower milk yields, longer CFSI and DO, and extended CCI values; suggesting reduced physiological and reproductive efficiency under summer calving conditions. In general, cows calving in cooler months tend to have higher 305-d milk yields and better reproductive performance, while summer calvings, particularly under hot/humid conditions, are associated with reduced production, more reproductive disorders, extended days open, and longer intervals to conception (Ray et al. 1992; Barash et al. 1996; Oseni et al. 2003; Van Eetvelde et al. 2020; Castro-Montoya et al. 2022). These monthly differences in reproductive performance seems consistent with seasonal patterns of heat stress exposure. The THI is widely used to quantify thermal stress, with values above 72 commonly considered indicative of conditions that negatively affect fertility (Armstrong 1994). As shown in Fig. 4, the months corresponding to autumn-winter calvings (April - August), coincide with the lowest number of days (averaged across 2010–2023) with THI exceeding the heat stress threshold (THI > 72). In contrast, spring/summer months (October–February), showed poorer reproductive indicators, were characterized by the highest frequency of days above this threshold. Thus, aligning calving patterns with the cooler season may optimize both productivity and fertility in pasture-based dairy systems in Argentina.

In Argentina, despite the introduction of genomically selected high-genetic-merit sires over the past decade, national average milk yield has remained stable over the past years (~5,900 kg/cow/year and 401 kg of milk solids/cow/year). While registered Holstein herds showed an increase of ~40% in milk production (from ~5,500 to ~7,900 kg/cow/year) between 1995 and 2015, this trend has plateaued during the last decade (Pardo et al. 2023).

Moreover, most improvements in Argentina have stemmed from environmental or management factors (>70%) (e.g., feed quality, health protocols), rather than sustained genetic progress (Pardo et al. 2023). Phenotypic trends for the unregistered Holstein analyzed in this study across the 2010–2023 period were consistent with an overall stabilization of performance, with modest positive slopes for adjusted milk yield and only limited improvements in fertility indicators. However, the improvement observed in milk yield and fertility traits during the most recent period (2020–2023) is noteworthy, particularly the steeper decline in DO and CCI. Similar trends have been reported in North American dairy populations, where greater emphasis has been placed on fertility and functional traits within selection indices (García-Ruiz et al. 2016; Cole and VanRaden 2018). It is possible that differences between selection environments and local production conditions, where nutritional supply and thermal stress differ substantially, influence the phenotypic performance observed in pasture-based herds. This interpretation aligns with previous evidence suggesting that breeding programs optimized for confined systems may be suboptimal for grazing-based dairy production (Hansen 1999), although these effects were not directly evaluated in the present study

An inefficient adaptation of imported germplasm to local production systems could contribute to genotype × environment interactions that influence productive performance limiting the full expression of genetic potential (Hayes et al. 2009). Therefore, there may be opportunities to develop breeding strategies in Argentine dairy systems tailored to the specific demands of pasture-based systems, that not only continue to select for milk yield, but also integrate traits relevant to animal welfare (health, fertility, longevity, etc) and long term environmental sustainability (e.g. feed efficiency, methane reduction).

## Data Availability

The authors do not have permission to share the datasets generated during and/or analysed during the current study.
